# Self-Help in Institutional Economy

**Published:** 1913-02-01

**Authors:** 


### SELF-HELP IN INSTITUTIONAL ECONOMY.

### V.—The Co-operative Laundry.

English and German hospital opinion decidedly recommends every large hospital to have its own laundry department, and thus to be independent of outside laundries. In accordance with this opinion, every modern hospital in these countries, which makes any pretensions at being first-class, has its own laundry, in which the plant is operated either by low-pressure steam or by electricity. The advantages of such a laundry department are now so well recognised that it argues an ignorance of hospital administration on the part of those who do not know them. The objections to the laundry department as an integral part of every hospital are, however, not so well known, and we may stop for a moment to deal with them. Stress is chiefly laid upon them by hospital workers in France and Belgium—to English, German, and American hospital authorities the advantages of the institutional laundry department far outweigh these theoretical and practical objections.

### Objections to the Laundry Department.

The establishment of a model laundry department, properly equipped and capable of dealing with a large amount of soiled linen in the best and most economic manner, is a very costly undertaking. Not only is it necessary to have elaborate disinfecting, sorting, washing, rinsing, drying, wringing, ironing, and folding apparatus, but in many cases it would also be necessary to have an elaborate water-softening plant. The laundry staff, too, is a comparatively large one, for although, by means of modern labour-saving machinery, washing by hand is no longer essential, every machine employed in a modern laundry needs an expert caretaker to look after it.

In the case of a small hospital it is obviously unjustifiable that all this expense should be incurred when the relatively small amount of soiled linen can be cleaned at an outside laundry. Even here, however, it is legitimate to resort to this argument, that a small hospital has not only soiled linen, but has infected linen as well, and that it is undesirable, and may even be dangerous, to send such linen for cleaning to a private or public laundry where other linen is cleaned. This question of disinfection, indeed, is one of the strongest (perhaps the strongest argument) arguments in favour of the special laundry department as an integral part of the central economic building of every hospital. It is premised, too, that if on account of the expense the provision of a laundry department is undesirable for a small hospital, it is unwise for a large one, since it is relatively cheaper to deal with large quantities of foul linen in a laundry equipped with modern machinery than to deal with smaller quantities. The French authorities, therefore, think it more economical to have co-operation in institutional laundry work. They estimate that the quantity of foul linen requiring cleaning may be calculated at the rate of 2 kilograms (by weight) per patient per day. A hospital of 500 beds will, therefore, have a daily laundry of 1,000 kilograms, or an average of more than eight tons a week.*

This is the scale on which the laundry plant is designed, though in many hospitals the plant has been designed to deal only with an average of 120 kilograms per 100 patients. A modern washing-machine, driven by low-pressure steam, and occupying an area of one square metre, is generally estimated as possessing a capacity to deal easily with 75 to 100 kilograms weight of foul linen per hour—a capacity of 620-800 kilos for an ordinary working day. When we add the other machinery, we find that for a laundry capable of dealing with a daily average of 800 kilos of foul linen—for a hospital, that is, of 400 beds—we require an area of nearly 300 square metres. By increasing the power of the plant, double that quantity of laundry can be dealt with without having to enlarge the building or materially increase the surface area. A laundry that caters for a daily average of 3,000 kilograms is relatively cheaper, per kilo washed, than one which deals with only 800 kilos per day. The economy is effected by dealing with large quantities with the same machinery, whereby only a slightly larger amount of coal or electric power is used. Further saving is made in various ways, chiefly in the matter

* This figure may seem an exaggeration to those who are accustomed to the old calculation on the basis of $\frac{1}{2}$ to $\frac{3}{4}$ kilo per patient. Laundries based on this scale—which, it is needless to say, is utterly insufficient—are still being established in hospitals that claim to be modern.

of water softening, consumption of soap, electric bleaching and ironing, both of which are cheaper when carried out on a large scale than when applied to a small quantity of laundry material.

Some Famous Co-operative Laundries.

These considerations have influenced the French and Belgian hospital authorities to embark on a scheme of co-operation in regard to laundry work. At Brussels a central laundry department, which caters for all the hospitals, has been established outside the city. A similar central laundry exists at Antwerp and at Louvain. At Paris a central laundry has been in existence for many years. In 1861 the amount of hospital laundry treated at Paris was 6,000,000 kilos annually, a total which clearly shows how grossly imperfect was the hospital administration in those days. In 1889 this total had risen to 12,000,000 kilos, in 1895 it was 16,000,000, and to-day it is nearer 24,000,000. This shows well how modern hospital requirements have influenced the laundry work, for the number of beds in Paris has not increased in proportion to this increase in the work of the laundry.

The central laundries in France and Belgium undoubtedly conduce to economy. A central staff, a central administration, and a central department combine to make the work of dealing with the huge output of foul linen, derived from all the hospitals, comparatively easy, since the system of dealing with it is so highly organised that everything goes smoothly and easily. The main difficulties appear to be encountered in the collection and distribution of the laundry. Mistakes are of constant occurrence, notwithstanding the care taken in arranging the daily lists. The work done at the central laundry is very excellent and in quality compares favourably with that executed in the best hospital department.

The cost, taking into account everything connected with the establishment, especially the relatively expensive distribution and collecting service and the salaries of the large staff of employees, is about 10 per cent. to 15 per cent. below that which obtains in most hospital laundries. In that way, therefore, the co-operative venture is justified, but, on the other hand, many hospital superintendents, who declare themselves quite contented with the system as it works at present, state that they should like to have a small private laundry attached to their institutions. This is in reality a confession that the co-operative system as applied to laundry work is not perfect. Especially where a hospital has a ward for infectious or contagious diseases, as is the case with nearly every foreign hospital, although not with English institutions, since in this country the necessity for special fever hospitals has been generally recognised, a private laundry attached to the institution is desirable. At the central laundry establishment an elaborate disinfecting department is in active operation, but the transport of the infected clothing and linen from the hospitals to the station require that the material should be disinfected at the institutions themselves. If not so disinfected they are carried through the streets at some risk; a method which is certainly not hygienic and which should not be allowed. This is one of the main objections to the central laundry station, but it is a very serious one and cannot be lightly disregarded.

In Russia an attempt has been made to establish co-operative hospital laundries, but it has not been a success. Most of the new Russian hospitals have excellent laundry departments, which are equipped and staffed in a model manner. Similar departments exist in the new German (a fine model being at the Moabit Hospital, Berlin), American (good models are seen at the Philadelphia Hospital and the German Hospital at Boston), Italian (at Bologna and Rome), Swiss (particularly at Berne), and one or two of the newer Austrian hospitals. In English hospitals the laundry department, where it exists, is generally one of the best and most up-to-date departments in the institution. The same may also be said for those Scottish institutions which have given special attention to their laundry work. On the other hand, the department is by no means good in Irish and Colonial hospitals, even in the more modern types.

